# The Training of Midwives

**Published:** 1886-11-20

**Authors:** 


### THE TRAINING OF MIDWIVES.

H. C. H. writes from 3, Hyde Street West, Clifton Hill, New Cross: "Will you kindly inform me if there is a Lying-in Hospital where I could learn midwifery gratis, or what is the smallest fee?

*** Will some of our readers answer this question? —Ed. T. H.

